# A Short Intervention and an Interactive e-Learning Module to Motivate Medical and Dental Students to Enlist as First Responders: Implementation Study

**DOI:** 10.2196/38508

**Published:** 2022-05-18

**Authors:** Victor Taramarcaz, Tara Herren, Eric Golay, Simon Regard, Sébastien Martin-Achard, Francois Mach, Nicolas Schnetzler, Gaëtan Ricci, Ido Zamberg, Robert Larribau, Marc Niquille, Mélanie Suppan, Eduardo Schiffer, Laurent Suppan

**Affiliations:** 1 Division of Emergency Medicine Department of Anesthesiology, Clinical Pharmacology, Intensive Care and Emergency Medicine University of Geneva Hospitals and Faculty of Medicine Geneva Switzerland; 2 Save a Life Swiss Emergency Responders Association Geneva Switzerland; 3 Cardiology Department University of Geneva Hospitals and Faculty of Medicine Geneva Switzerland; 4 Division of Anesthesiology Department of Anesthesiology, Clinical Pharmacology, Intensive Care and Emergency Medicine University of Geneva Hospitals and Faculty of Medicine Geneva Switzerland

**Keywords:** basic life support, cardiopulmonary resuscitation, first responder, undergraduate medical education, out-of-hospital cardiac arrest, medical education, e-learning, digital education, medical student, blended learning

## Abstract

**Background:**

Prompt and proficient basic life support (BLS) maneuvers are essential to increasing the odds of survival after out-of-hospital cardiac arrest. However, significant time can elapse before the arrival of professional rescuers. To decrease these delays, many countries have developed first responder networks. These networks are composed of BLS-certified lay or professional rescuers who can be dispatched by emergency medical communication centers to take care of those who experience out-of-hospital cardiac arrest. Many systems are, however, limited by a relatively low number of active first responders, and first-year medical and dental students may represent an almost untapped pool of potential rescuers. On top of providing an enhanced BLS coverage to the population, this could also help medical students be better prepared to their future role as certified health care providers and address societal expectations regarding health care students.

**Objective:**

Our objective was to describe the impact of a short motivational intervention followed by a blended BLS course (e-learning and practice session) designed to motivate first-year medical and dental students to enlist as first responders.

**Methods:**

A short, web-based, motivational intervention presenting this project took place, and first-year University of Geneva, Faculty of Medicine students were provided with a link to the study platform. Those who agreed to participate were redirected to a demographic questionnaire before registering on the platform. The participants were then asked to answer a second questionnaire designed to determine their baseline knowledge prior to following an interactive e-learning module. Upon completion, a web-based booking form enabling them to register for a 1-hour practice session was displayed. These sessions were held by senior medical students who had been trained and certified as BLS instructors. The participants who attended these practice sessions were asked to answer a postcourse questionnaire before receiving the certificate enabling them to register as first responders.

**Results:**

Out of the 529 first-year students registered at University of Geneva, Faculty of Medicine on January 14, 2021, 190 (35.9%) initially agreed to participate. Moreover, 102 (19.3%) attended the practice sessions, and 48 (9.1%) had completed all training and enlisted as first responders on the dedicated platform, Save a Life, at 6 months (July 14, 2021). Postcourse confidence in resuscitation skills was associated with a higher likelihood of registering as first responder (*P*=.03). No association was found between prior BLS knowledge and the probability of registering to a practice session (*P*=.59), of obtaining a course completion certificate (*P*=.29), or of enlisting as first responder (*P*=.56).

**Conclusions:**

This study shows that a motivational intervention associated with a short BLS course can convince medical students to enlist as first responders. Further studies are needed to understand the rather low proportion of medical students finally registering as first responders.

**International Registered Report Identifier (IRRID):**

RR2-10.2196/24664

## Introduction

### Background

According to the World Health Organization, ischemic heart disease is the current leading cause of death worldwide [1], and most out-of-hospital cardiac arrests (OHCAs) are linked to this condition [2]. Survival and neurological outcomes greatly depend upon the prompt provision of basic life support (BLS) maneuvers and on the availability of automatic external defibrillators (AED) [3-6]. In Geneva, Switzerland, BLS maneuvers were only provided in 40% of OHCA cases between 2009 and 2012, according to a retrospective study published in 2018 [7]. This proportion is lower than that found on average in Europe [2], and there is, consequently, considerable room for improvement.

To enable the provision of BLS maneuvers prior to the arrival of professional rescuers, first responder systems have been created in many regions of the world. These systems allow emergency medical dispatchers to send adequately trained individuals to take care of OHCA situations before the arrival of professional help. Although different technological solutions have been developed, some of which are currently used in Switzerland, most operate according to the same principles. In general, first responders receive a notification on their smartphone through a specific app. The ones who are both available and within a 3-km radius of the scene receive the exact coordinates after accepting the mission. This allows a timely provision of BLS maneuvers by adequately trained responders and, consequently, higher survival rates [8-12]. The major functional limitation of these platforms is related to the number of registered first responders, their location, and their availability. By raising the sheer number of first responders, a country or state could increase its overall coverage and henceforth improve OHCA outcomes. In Switzerland, the state of Ticino was first to launch a first responder system in 2005 [13]. Since then, other systems have been developed throughout the country, and most of the 26 Swiss states now run their own first responder organization [14]. In Geneva, the first responder platform, *Save a Life,* enables any adult in possession of a valid BLS-AED certificate to register as first responder [15]. Recently created, *Save a Life* counted only 260 active first responders in 2019, its first year of activity [16].

Medical students may represent an almost untapped pool of potential first responders. Previous studies have shown that first-year medical students feel that they would be expected to act in case of an emergency but feel unprepared to face OHCA situations [17]. This lack of confidence seems legitimate as their BLS knowledge is not much more advanced than that of the general population [4]. Giving these novice students the opportunity to gain solid knowledge about first aid procedures could increase their confidence, motivating them to join a first responder platform and thereby potentially increasing the rate of BLS maneuvers initiated prior to the arrival of professional rescuers.

### Objective

The main objective of this study was to describe the impact of implementing a short motivational intervention and a blended learning path designed to motivate first-year medical and dental students to enlist as first responders. To be able to enlist as first responders, students were required to follow an e-learning module, attend a practice session, and answer multiple questionnaires.

## Methods

### Study Design

This was a prospective implementation study based on a research protocol published on November 6, 2020 [18]. When relevant, methods and results are reported according to the Checklist for Reporting Results of Internet E-Surveys [19].

### Ethical Considerations

A declaration of “no objection” was issued by the local ethics committee (Req-2020-01143) as this project did not fall within the scope of the Swiss Federal Act on Research involving Human Beings [20]. This project was also approved by the vice dean of undergraduate education at the University of Geneva, Faculty of Medicine (UGFM).

### Participants and Enrollment

The target population consisted in a convenience sample including all UGFM first-year medical and dental students. Those who were already registered as first responders were excluded. No financial incentive was given to promote participation. The whole learning path was completely free, and students were informed that they would be granted a specific BLS-AED certificate upon a successful completion of the training program. This certificate had a 1-year validity and only allowed them to register on the *Save a Life* platform.

Two senior medical students presented the project to all these students on January 14, 2021. The presentation, which was originally intended to take place live in an auditorium at the beginning of a lecture about atherosclerosis, was held online because of restrictions linked to the COVID-19 pandemic. It was broadcasted as part of this lecture through the university’s web-based platform on which all courses could be followed live or on-demand. The presentation included an overview of the project and the learning path, an estimate of its duration, and a presentation of the *Save a Life* first responder system [15]*.* The URL linking to the study site was also shown on the last slide of the presentation. The study URL was also sent to all medical and dental students through a mailing list a few hours after the presentation (Multimedia Appendix 1). Theoretically, this official UGFM mailing list should have included all first-year medical and dental students. However, subscribing to this list was not mandatory, and the university’s policy allows anyone in possession of a University of Geneva email address to subscribe to any mailing list. This made it impossible to ascertain that the whole population could be reached. A second and last email reminder promoting registration was sent to the whole mailing list on March 8, 2021.

### Web-Based Platform

A specific web-based study platform was developed using the Joomla 3.9 [21] content management system (Open Source Matters). This platform hosted the questionnaires, the e-learning module, and was used to manage the registration process for the practice sessions. All questionnaires were created using Community Surveys 5.5 (CoreJoomla). Three authors thoroughly tested the platform prior to study inception. All collected data were stored on an encrypted MySQL database (MariaDB 5.5.5) located on a Swiss server and handled in accordance with the European General Data Protection Regulation [22].

### Consent and Initial Questionnaire

The URL provided to the students led to an introductory page designed to determine whether they were already registered as first responders, which was the first of the two exclusion criteria (Table 1 and Multimedia Appendix 2). Those who met this first criterion were nevertheless given the possibility of following the e-learning module. All the other participants were redirected to a form designed to gather their informed consent and determine whether our second and last exclusion criteria were met (ie, not being part of the target population). A disclaimer (Multimedia Appendix 3) detailing the study design and data handling procedures was displayed at the top of the main page, and participants had the possibility of reading and downloading a comprehensive 3-page document (Multimedia Appendix 4) including additional details. The contact information of 4 investigators was provided to enable them to directly ask further questions.

Students who refused to participate were prompted to give a reason and were nevertheless allowed to access the e-learning module (Table 1 and Multimedia Appendix 2). They were offered the possibility of following the practice sessions upon completion of the module and could therefore obtain a course completion certificate and join the first responder system regardless of their willingness to participate in the study.

Those who did not meet the exclusion criteria and agreed to participate were redirected to a short registration form, which was composed of 3 fields only: first name, last name, and email address. The students’ identities were collected to allow the creation of nominative certificates. Email addresses were used to directly contact the participants, give them information regarding the practice sessions, and send them their BLS certificate provided they had successfully completed the whole learning path. The participants were informed that they could withdraw from the study at any time.

After registration, the participants were asked to fill out a precourse questionnaire (Table 2 and Multimedia Appendix 5) designed to gather demographic data and assess initial BLS-AED knowledge. The questionnaire was adapted from a study by Sturny et al [4] and based on the 2015 International Consensus on Cardiopulmonary Resuscitation and Emergency Cardiovascular Care Science With Treatment Recommendations [6].

**Table 1:** Screening questionnaire and consent form (survey page number 1).

Survey field and question		Type of question
**Already filled the questionnaire or exclusion criteria**		
	Already a first responder?	Yes/no
**Demographics**		
	Student at UGFM^a^?	Yes/no
	If no: current professional status?	Open
**Consent**		
	Agree to participate?	Yes/no
	If no: reasons for refusal?	MAQ^b^
	If no: access to the e-learning module?	Yes/no

^a^UGFM: University of Geneva, Faculty of Medicine.

^b^MAQ: multiple answer question.

**Table 2:** Precourse questionnaire.

Survey page, field, and question		Type of question
**1—Demographics**		
	Year of birth	Open (Regex^a^)
	Gender	MCQ^b^
	Medical, biomedical, or dental medicine student	MCQ
	Former student or graduate of another health care profession	MCQ
	Target specialty	MCQ
**2—General BLS^c^** **knowledge**		
	Ever heard of BLS or ACLS^d^ before	Yes/no
	Meaning of AED^e,f^	Open
	Year of the last BLS guidelines update	Open (Regex)
	Phone number of the emergency medical communication center^f,g^	Open
**3—Prior BLS experience**		
	Prior BLS training	MAQ^h^
	Wish for additional BLS training	Yes/no
**4—Specific BLS knowledge**		
	Criteria used to recognize OHCA^f,g,i^	MAQ
	BLS-sequence^f,g^	Ordering
	Artery for pulse assessment^f^	MCQ
	Compression depth^f,g^	MCQ
	Compressions: ventilation ratio^f^	MCQ
	Compression rate^f,g^	MCQ
	Compression-only CPR^f,g,j^	Yes/no
	Foreign body airway obstruction^f^	MCQ

^a^A Regex validation rule was used to avoid invalid entries.

^b^MCQ: multiple choice question (only one answer accepted).

^c^BLS: basic life support.

^d^ACLS: advanced cardiovascular life support.

^e^AED: automatic external defibrillator.

^f^Items used to calculate the 10-point score (initial BLS knowledge).

^g^Items used to calculate the 6-point score (essential BLS knowledge).

^h^MAQ: multiple answer question (more than one answer accepted).

^i^OHCA: out-of-hospital cardiac arrest.

^j^CPR: cardiopulmonary resuscitation.

### e-Learning and Practice Sessions

After completing this questionnaire, participants were granted access to an interactive e-learning module developed under Storyline 3 (Articulate Global, LLC). This e-learning was adapted from a similar module used to teach BLS-AED procedures to second-year UGFM students. It was designed to last 30 minutes, but no time limit was set for its completion.

The objectives of this e-learning module were designed according to the Swiss Resuscitation Council guidelines for the training of BLS-AED providers [23] and adapted according to the COVID-19 pandemic guidelines [24]. The goal of this module was to enable students to (1) identify a cardiorespiratory arrest, (2) recall the emergency numbers and alert professional help, (3) identify threats to their own safety, (4) acquire the knowledge necessary to perform high-quality cardiopulmonary resuscitation (CPR), and (5) use an AED. To ensure an optimal focus, all these subjects were presented in an interactive way.

After completing the e-learning module, the participants were able to register for the practice sessions. These sessions lasted 1 hour and were limited to 4 participants according to the regional COVID-19 regulations in effect at the time [25,26]. Seventeen senior medical students acted as instructors during these sessions. For the purpose of this study, these senior students had been trained and certified as BLS-AED instructors according to the Swiss Resuscitation Council guidelines between January and March 2021. Owing to the availability of training rooms and instructors, a total of 128 training slots were available between February 8, 2021, and April 30, 2021. The instructors were provided with a comprehensive checklist to ensure that all critical points had been covered and mastered by the participants even though the actual structure of the session was left at their discretion.

### Final Questionnaire and Certification

The participants who successfully completed the practice sessions were sent an email inviting them to fill a postcourse questionnaire (Table 3 and Multimedia Appendix 6). A course completion certificate was delivered to all the participants who completed this last questionnaire.

**Table 3:** Postcourse questionnaire.

Survey page, field, and question		Type of question
**1—Opinion**		
	Appreciation	Yes/no
	If yes: positive thoughts	MAQ^a^
	If no: negative thoughts	MAQ
	General comments	Free text
**2—Confidence**		
	Precourse confidence for OHCA^b^ management	Likert scale (1-5)
	Postcourse confidence for OHCA management	Likert scale (1-5)
	Factors contributing to confidence	Likert scale (1-5)
	Factors contributing to lack of confidence	Likert scale (1-5)
	Other comments on confidence	Free text
**3—First responders**		
	Intention to register as first responder	Yes/no
	If yes: contributing factors	Likert scale (1-5)
	If no: impeding factors	Likert scale (1-5)
	Other factors	Free text
**4—Improvement**		
	Suggestion for improvement	Free text

^a^MAQ: multiple answer question.

^b^OHCA: out of hospital cardiac arrest.

### Deviations From the Research Protocol

The research protocol had to be adapted to cope with unforeseen realities. First, since the course during which the study was presented was web-based, a QR code was not created, and a short URL was displayed. Second, Joomla 3.10 had not been released at the time this study started, and the platform was developed under Joomla 3.9. In addition, we added a postcourse questionnaire after the publication of the research protocol to evaluate the participants’ confidence and gather information regarding the practice sessions. Therefore, minor modifications were made to the second questionnaire for consistency purposes. Furthermore, in line with the previous study by Sturny et al [4], we computed a 6-point score focusing on “essential BLS-knowledge.” Finally, rather than using already-trained BLS instructors, we decided to specifically train senior medical students for that purpose.

### Outcomes

The primary outcome was the number of first-year students who had enlisted as first responders on the *Save a Life* platform by July 14, 2021 (ie, exactly 6 months after the course presenting our study was held). Secondary outcomes were the number of participants who agreed to participate, registered on the platform, began the e-learning module, completed this module, registered for practice sessions, attended these sessions, and obtained a certificate. The evolution of their confidence and the association of postcourse confidence with the probability of registering on the *Save a Life* platform were also analyzed.

Even though the web-based platform was thoroughly tested before study inception, we could not rule out the occurrence of technical difficulties. Therefore, all technical difficulties were recorded and reviewed. Free comments were also analyzed.

### Statistical Analysis

Data were extracted to comma-separated value files, and Stata 17.0 (StataCorp LLC) was used for data curation and statistical analysis. The curated data file is available as Multimedia Appendix 7. The different proportions of students are presented using descriptive statistics (n [%]). The items used to calculate the prior BLS knowledge scores can be found in Table 2. Each item was worth 1 point, with no differential weighting. After assessing the normality (first graphically, then using the Kolmogorov-Smirnov test), Student *t* test was used to determine whether there was a difference in the interest in further training according to prior BLS knowledge (using both the 10-point and 6-point scores). The same test was used to assess the probability of obtaining a certificate and of registering as first responder according to prior BLS knowledge. The Student *t* test was also used to describe whether postcourse confidence was different between those who enlisted as first responders and those who did not. A paired *t* test was performed to assess the evolution of student confidence before and after the course. Factors reported as affecting confidence in resuscitation skills and motivation to enlist as first responders according to the questions based on the 5-point Likert scales are described graphically. The preplanned sensitivity analysis—which was designed to determine if differences existed between the students who immediately accepted to participate and those who rallied the study after following the e-learning module—was not performed as there were no participants in this latter group. A *P* value of less than .05 was considered significant.

In addition to the preplanned analyses, we performed a qualitative analysis of the comments obtained through the postcourse questionnaire. The participants were able to leave free comments in 4 different sections (Table 3). Because responses were sometimes entered in inappropriate sections, all comments were pooled before being sorted by theme. Representative answers were translated using DeepL Translator (DeepL) and added to the results section. We also analyzed the emails sent by medical students regarding the technical difficulties they had encountered.

## Results

**Figure 1:**
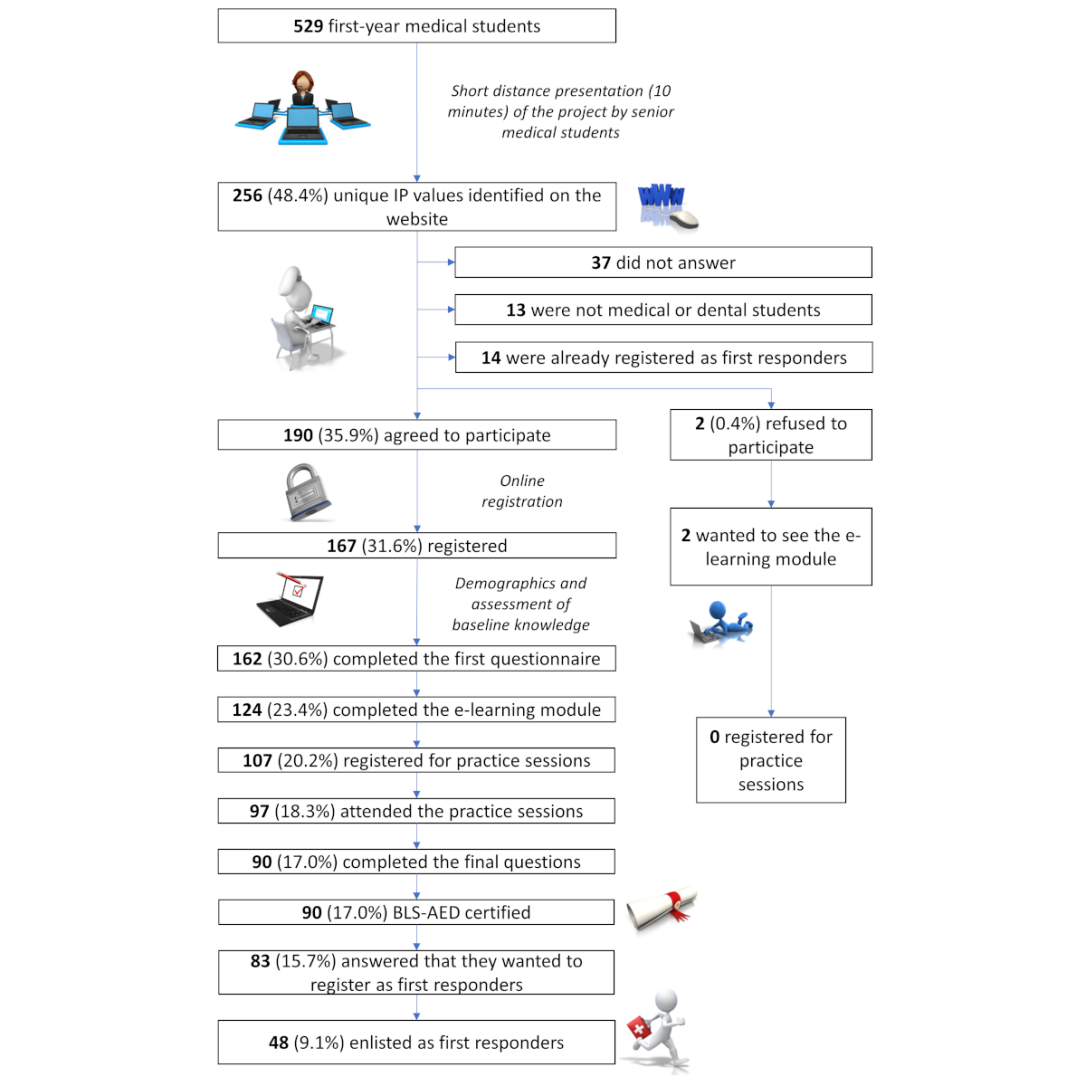
Study flowchart. AED: automatic external defibrillator; BLS: basic life support.

A total of 529 medical and dental students were registered at UGFM at the time this study started (8 more than that stated in the original study protocol). Figure 1 shows the different steps and the associated proportions. By July 14, 2021, 48/529 (9.1%) students had completed the whole process and enlisted as first responders on the *Save a Life* platform. This was a slightly lower proportion than initially expected according to the original protocol (10%, 53/529) [18].

The characteristics of the 162/529 (30.6%) students who completed the first questionnaire are detailed in Table 4. The respondents unanimously answered that they wished for more BLS training. No association was found between prior BLS knowledge and the probability of registering to a practice session (*P*=.59), of obtaining a course completion certificate (*P*=.29), or of enlisting as first responder on the *Save a Life* platform (*P*=.56). The proportion of dental students who completed the first questionnaire was significantly lower than that of medical students (7/55, 12.7% vs 155/474, 32.7%; *P*=.002).

Of the 124 participants who completed the e-learning module, 28 (22.6%) reported problems with the component used to register for practice sessions. Help was provided by email, and all problems were solved manually by the study team. Moreover, 10/107 (9.3%) students who had registered for a practice session did not show up. They were all contacted to determine the reason preventing them from attending the practice sessions, but none of them answered our request for information.

**Figure 2:**
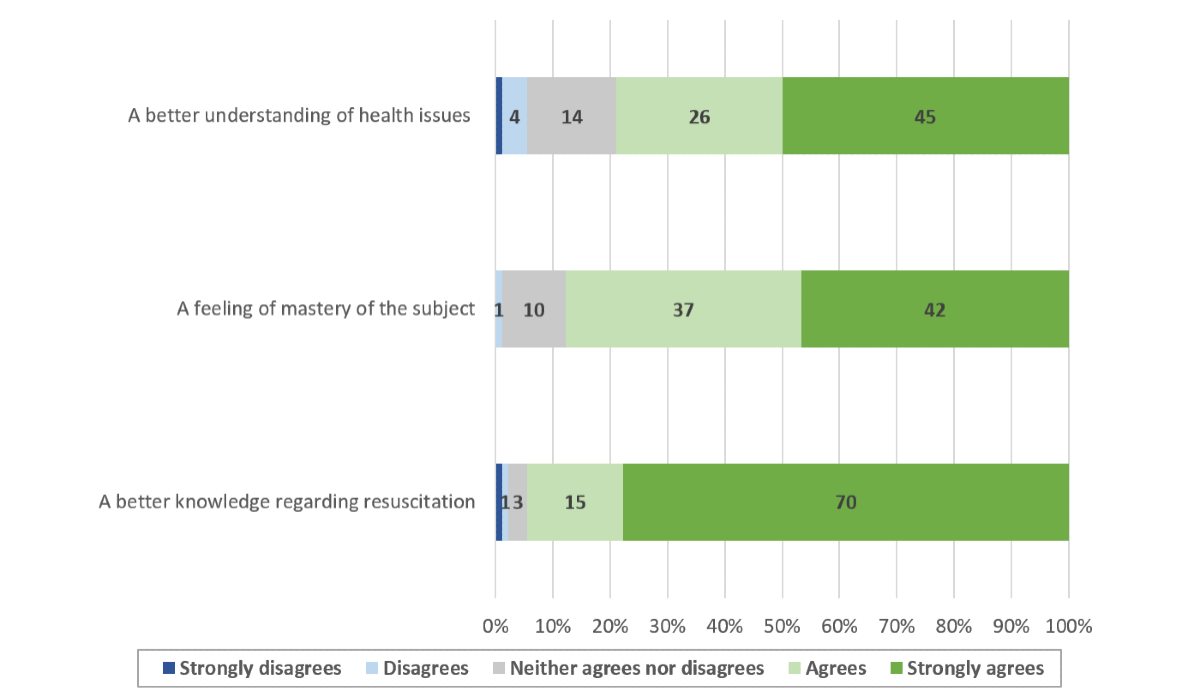
Factors contributing to an increased confidence in resuscitation skills.

**Figure 3:**
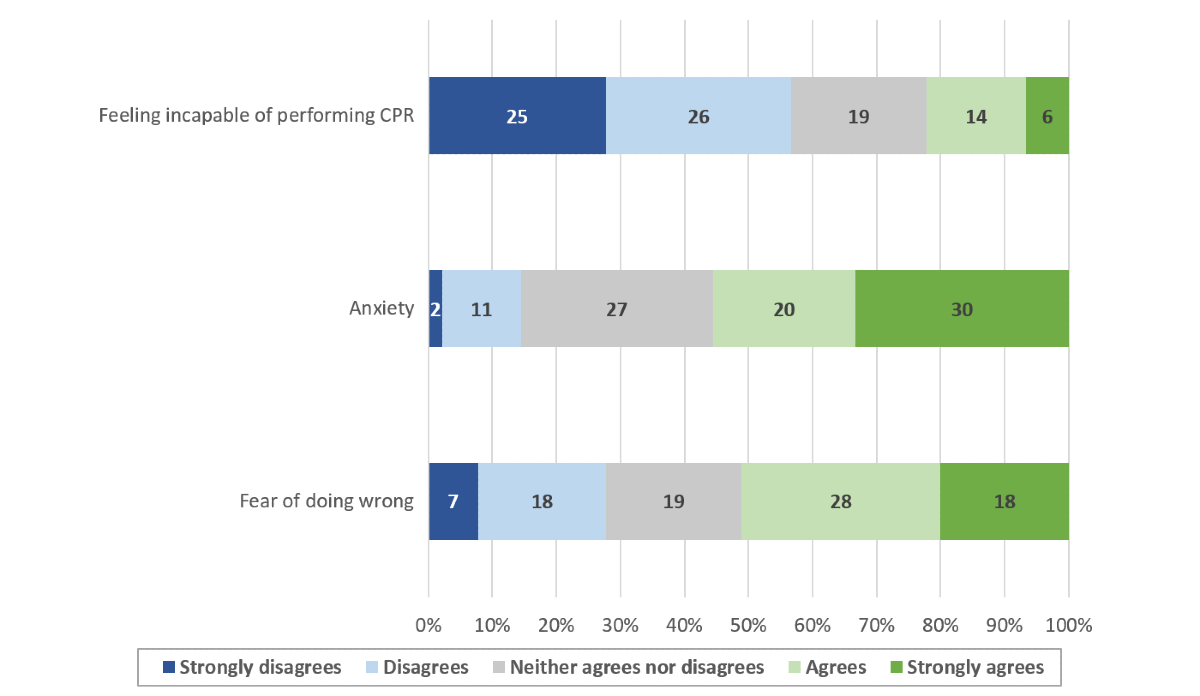
Factors limiting student confidence in resuscitation skills. CPR: cardiopulmonary resuscitation.

**Figure 4:**
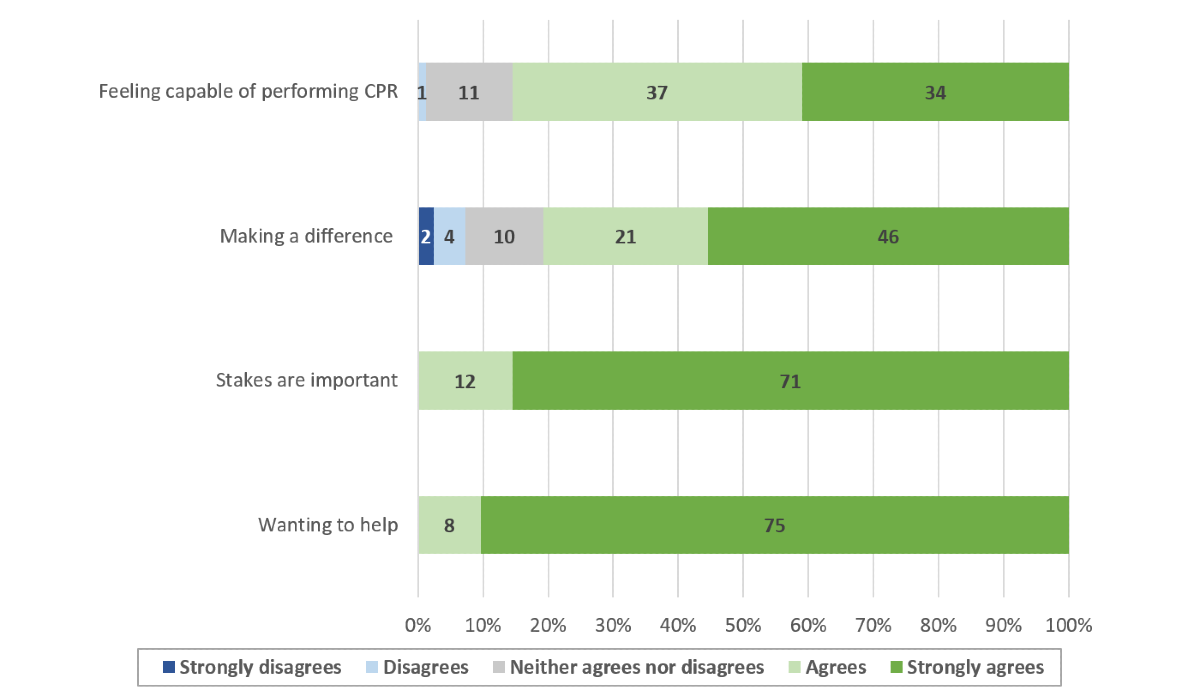
Factors motivating students to enlist as first responders. CPR: cardiopulmonary resuscitation.

**Figure 5:**
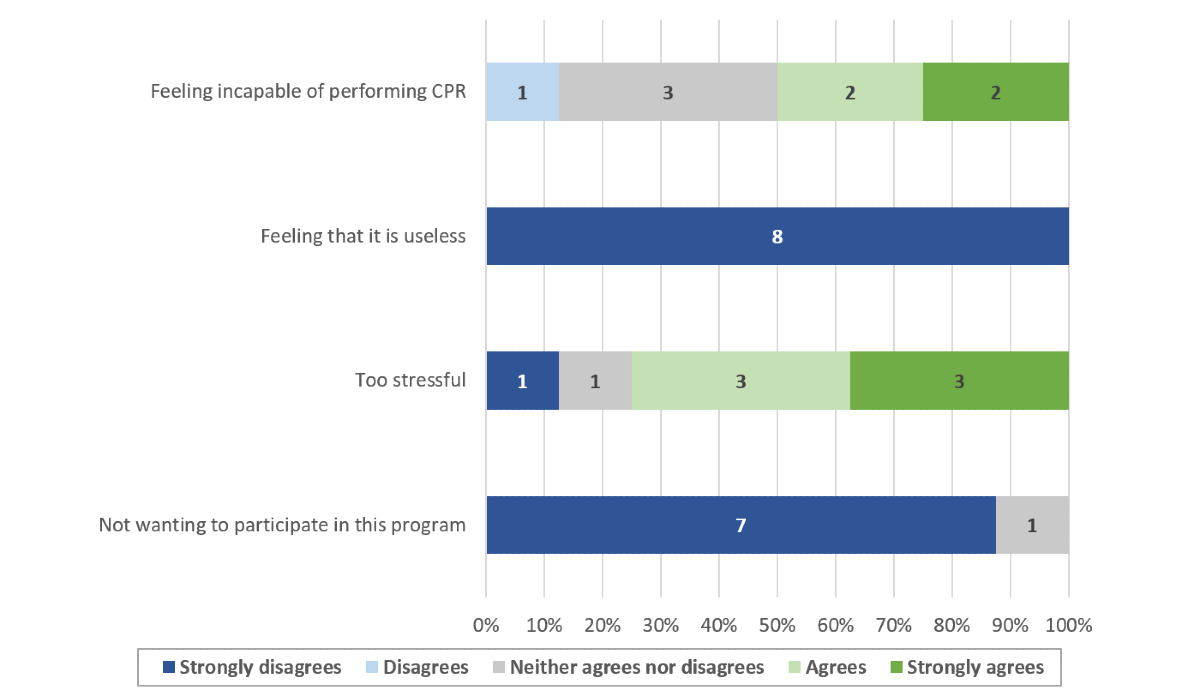
Factors preventing students from enlisting as first responders. CPR: cardiopulmonary resuscitation.

Most of those who attended the practice sessions answered our postcourse questionnaire (90/97, 92.8%). There was a significant increase in confidence regarding OHCA management skills after following the learning path (4.2, SD 0.6 vs 2.1, SD 0.9; *P*<.001). The majority of the students who answered the postcourse questionnaire reported that they were willing to sign up as first responders (83/90, 92.2%). Factors affecting student confidence and their motivation to enlist as first responders are shown in Figures 2-5.

Those who felt more confident after the course were more likely to register as first responders (registrants had a mean confidence of 4.3, SD 0.5 vs 4.0, SD 0.6; *P*=.03).

All the students who completed the postcourse questionnaire had a positive opinion of the learning path (90/90, 100%) and all of them would have recommended this course to other first-year students. Moreover, 30/90 (33.3%) students left a total of 40 comments in the dedicated sections of the postcourse questionnaire. Most were positive feedbacks regarding the project (29/40, 72.5%). Three other themes were identified, which were course duration, integration of the course in the standard curriculum, and availability of a face-to-face course during the COVID-19 pandemic. Many students (15/40, 37.5%) thought that the course was too short and that there was not enough time for hands-on practice. One student commented, “1 hour is too short! Sessions scheduled for a little longer (1h30-2h) would allow us to be less stressed by time and to practice better.“ Some students (5/40, 12.5%) considered that such a course should be mandatory during the first year of their curriculum. Finally, a few students (3/40, 7.5 %) declared that attending this course had been a unique occasion to have face-to-face training during the COVID-19 pandemic. A student commented that “It was really super interesting and rewarding!!! And it was really nice to be able to do some practice,” while another wrote that it was “A pleasure to see people in these times.”

**Table 4:** Characteristics of the 162 participants who answered the first questionnaire.

Characteristics		Enlisted as FR^a^ (n=48)	Did not enlist as FR (n=114)	*P* value
Age (years), mean (SD)		20.3 (4.1)	20.1 (4.7)	.88
**Gender, n (%)**				.71
	Male	14 (29.2)	30 (26.3)	
	Female	34 (70.1)	84 (73.7)	
	Other	0 (0)	0 (0)	
**Curriculum**, **n (%)**				.95
	Medical	46 (95.8)	109 (95.6)	
	Dental	2 (4.2)	5 (4.4)	
**Knowledge score, mean (SD)**				
	10-point score	5.1 (1.7)	4.9 (1.6)	.56
	6-point score	2.6 (1.0)	2.4 (1.1)	.19
Already followed a BLS^b^ course, n (%)		38 (79.2)	80 (70.2)	.24
Wishes for more BLS training, n (%)		48 (100)	114 (100)	N/A^c^

^a^FR: first responder.

^b^BLS: basic life support.

^c^N/A: not applicable.

## Discussion

### Main Considerations

This implementation study shows that less than 9.1% (n=48) of first-year UGFM medical and dental students enlisted as first responders after following a short motivational intervention and being offered the opportunity of following a blended learning path including an asynchronous e-learning module and hands-on practice. This proportion was slightly lower than the 10% target we had aimed at according to our original protocol [18].

Numerous reasons might explain this lower-than-expected result. First, even though the presentation was a success with almost half the promotion connecting to the platform, it took place rather late in the academic year, and practice sessions were scheduled even later. Therefore, because these junior students are under considerable pressure given the high failure rate, many might have elected to prioritize their revisions for the final exams over joining the first responder system regardless of their interest. Future studies will need to assess whether earlier inception can lead to higher registration rates. Second, while we strived to shorten the practice sessions in an attempt to increase the participation rate, some students might have felt that these sessions were too short to allow them to master BLS procedures. This might have prevented some participants from registering as first responders. Finally, a rather high proportion of students experienced technical difficulties with the registration component. Despite the help provided by email, this issue may have increased attrition at this stage.

The restrictions linked to the COVID-19 pandemic context may have played a role in our study. As all faculty courses were web-based, our first intervention was aired on the university streaming platform allowing students to skip it at will. This format also prevented us from interacting with the students and from answering their questions right away, and all further communications were carried out by email. While these elements might have contributed to a lower participation rate, the fact that we offered one of the few face-to-face courses might have played in our favor. Indeed, some comments showed that the students who participated suffered from a lack of social interaction and felt that the practice session was an almost unique occasion to discover the university premises and to directly talk to their peers. To assess the impact of these changes in the academic curriculum and given the relatively high enthusiasm showed by some students, this learning path will be offered once again during the academic year 2021-2022.

The confidence of the participants regarding their resuscitation skills was significantly improved, and the contributing factor most often reported by the students was a better knowledge of the subject. In our study, a higher confidence was associated with a higher probability of registering as first responder. However, less than two thirds of the students who completed the whole training process and obtained a course completion certificate had enlisted as first responders at 6 months. Interventions further strengthening student confidence in their resuscitation skills could help increase this proportion, and their impact should be assessed through further studies. Nevertheless, since people who feel more confident in their abilities are also more likely to act when faced with a stressful emergency [27], the participants who decided not to enlist as first responders might still be more likely to perform CPR if needed after following the learning path. This could prove particularly valuable since many studies show that BLS knowledge and skills are often limited in health care workers and students. Furthermore, starting resuscitation courses sooner during the medical curriculum could be advantageous since resuscitation skills tend to improve according to the number of BLS training sessions [28,29]. In addition, the majority of students who reported wanting to enroll as first responders cited a desire to help as a main contributing factor. If the benefits of teaching BLS-AED courses during the first year of medicine curriculum could have an important impact on public health, generalizing this principle to mandatory school could have an even bigger one. Taking a step back from health care students, the European Resuscitation Council (ERC) recommends teaching CPR to children, preferably before the age of 12 years, in order to create long-lasting psychomotor skills and remember a short sequence of action over time. By doing so, targeting schoolchildren would be a way to increase the number of bystanders capable of performing CPR before the arrival of professional help and decrease the time of no flow [30].

In the past few years, the development of web-based courses has been expanding quickly, and the COVID-19 pandemic has increased this phenomenon even further [31]. Given the high potential number of interested students, the limited time available in the schedule of first-year medical and dental students, and the context of the COVID-19 pandemic, we believe that using an asynchronous distance e-learning module for the theoretical part of the course was particularly appropriate. Many studies have compared traditional didactic methods with e-learning interventions, the main limit being the huge variability of e-learning formats [32-34]. Among other parameters, interactivity and gamification mechanisms have been shown to affect the impact of e-learning interventions. Interactive e-learnings and serious games have been used in a few studies in Geneva and have demonstrated their superiority to the control group using usual course format [35,36]. While such modules have been shown to be useful to teach CPR procedures and decrease the time spent in workshops, they cannot replace hands-on practice [37].

### Limitations

Apart from our main limitation (ie, the adaptation of our study to the pandemic context with the subsequent limited interaction with first-year UGFM students), other limitations must be acknowledged. First and foremost, our design did not include a comparator, the lack of which prevented us from determining whether a certain type of motivational intervention would have been more effective than another. In addition, the type of learning path could also influence the intention of registering as first responder. Nevertheless, this study was carried out in accordance with our original protocol, and the limited number of first-year UGFM students would have limited the power of our study had a comparator been included. Moreover, 6 months represent a rather long delay between the initial intervention and the assessment of the number of UGFM students who had enlisted as first responders. However, more than 3 months had elapsed between the initial intervention and the last practice sessions, and it is improbable that elements other than our learning path would have prompted medical students to enlist as first responders given the high workload associated with their end-of-year exams, which were scheduled less than 2 months after the last practice session had been completed.

There is also bias in the answers to the questionnaires as the students participating in the study were interested beforehand and could not fully represent the knowledge of the whole promotion. Furthermore, we asked the participants about their confidence during the postcourse questionnaire, which is a recollection bias, as the students may have had a misperception of their confidence, overestimating or underestimating their abilities.

### Perspectives

Our initiative is in line with the concept of “systems saving lives” developed by the ERC [38]. Promoting and perpetuating such initiatives should help achieve the recommended number of available first responders, which is 10/km^2^ according to ERC guidelines [38,39]. To further increase the awareness of medical and dental students regarding the importance of BLS-AED procedures, 2 studies regarding resuscitation skills of medical students will be conducted during the academic year 2021-2022 at UGFM. The first one will evaluate the impact of our intervention on BLS knowledge in second-year medical students. The second study will follow a very similar protocol as the present one and assess the effect of the intervention after addressing the weaknesses identified. Therefore, the questionnaires will be slightly modified, and the motivational intervention will take place sooner in the academic year to avoid interference with final revisions and exams. The effect of less-constraining COVID-19 restrictions on participation will be assessed in the course of this study.

### Conclusion

After following a short motivational intervention, less than 10% of first-year medical and dental students enlisted as first responders after completing a blended learning path including an asynchronous e-learning module and hands-on practice. Including these future health care professionals in the first aid system early in their career and increasing the sheer number of potentially available first responders could help enhance survival and neurological outcomes in those having a cardiac arrest and participate in the building of their professional identity as a secondary benefit. Further studies are needed to understand the low proportion of medical and dental students finally enlisting as first responders and to determine whether different or additional teaching methods could increase this proportion.

## Appendix

Multimedia Appendix 1 Email sent to all first-year students.

Multimedia Appendix 2 Screening questionnaire and consent form.

Multimedia Appendix 3 Disclaimer on the website's main page.

Multimedia Appendix 4 Additional information regarding the study.

Multimedia Appendix 5 Precourse questionnaire.

Multimedia Appendix 6 Postcourse questionnaire.

Multimedia Appendix 7 Original data (in CSV and DTA formats).

## Abbreviations

AED: automatic external defibrillator
BLS: basic life support
CPR: cardiopulmonary resuscitation
ERC: European Resuscitation Council
OHCA: out-of-hospital cardiac arrest
UGFM: University of Geneva, Faculty of Medicine
