# Supplementary material for: A Short Intervention and an Interactive e-Learning Module to Motivate Medical and Dental Students to Enlist as First Responders: Implementation Study
Source: J Med Internet Res. 2022 May 18;24(5):e38508. doi: 10.2196/38508 (PMC9161047; doi:10.2196/38508)
Supplement: Multimedia Appendix 1 [file jmir_v24i5e38508_app1.pdf]

Multimedia appendix 1 – email sent to all first-year students

Original (French) version :

‼Concerne les premières années‼

Salut à toutes et à tous,

Vous étiez présent.e.s ce matin durant le cours du Pr Mach ?
Vous n'y étiez pas est le cours et encore dans votre liste des "à voir" sur médiaserveur ?

En tout les cas, une courte intervention a eu lieue ce matin avant le cours *Athérosclérose 5* et **elle vous concerne ! Nous vous invitons d'ailleurs à l'écouter si ce n'est pas déjà fait !**
Pour la toute première fois, l'association CPR de l'AEMG propose dans le cadre d'un travail de master et exclusivement aux premières années de la faculté un cours BLS-AED et l'occasion de rejoindre en avant première le système de soins genevois.

Attends Jamie, tu vas trop vite j'ai pas tout compris...

Voici le programme :
- Un **mini quizz** sur tes connaissances en premiers secours, dont les données anonymisées promettront de faire faire avancer la recherche.
- Un **E-learning de 30mn** pour remettre tout ça à jour
- Un fois l'E-learning complété vous pourrez vous inscrire pour **une séance de pratique** d'une heure (tu ne rêves pas, même en temps de COVID, on s'est arrangé pour vous proposer une séance de pratique en live, avec de vraies personnes !)
- Et si vous complétez l'E-learning et la formation, vous **recevrez un certificat en BLS-AED** qui vous permettra de vous **inscrire sur le réseau "Save a Life"** et ainsi rejoindre en avant première le système de soin genevois !

Le tout est disponible à l'adresse suivante : https://bls.anesth.ch
Pour des questions : bls-aemg@unige.ch

Pourquoi participer ?

- Ce sera ta seule occasion de faire de la pratique de l'année !
- Tu vas découvrir en avant première le monde des urgences
- Pour vanner tes ami.e.x.s dans les années supérieures car tu seras mieux formé en réanimation qu'eux
- La formation est courte
- Le cours est donné par des étudiants des années supérieures et se faire un réseau ne fait jamais de mal
- Tu recevras un joli papier qui en plus d'être utile, est aussi utile sur ton CV
- Pour enfin avoir une raison d'enfiler un pantalon et sortir de chez toi

En gros, cliquez sur ce lien (https://bls.anesth.ch), parcourez l'E-learning et venez apprendre à sauvez des vies !

Nous nous réjouissons de vous voir,

Victor Taramarcaz & Tara Herren

Translated version using DeepL

Concerning the first years

Hi everyone,

Were you present this morning during Prof Mach's lecture?
You were not there is the lecture and still in your "to see" list on media server?

In any case, a short intervention took place this morning before the Atherosclerosis 5 **course and it concerns you! We invite you to listen to it if you haven't already done so!**
For the very first time, the CPR association of the AEMG is offering a BLS-AED course as part of a master's thesis and exclusively to the first years of the faculty.

Wait Jamie, you're going too fast, I didn't understand everything...

Here is the program:
- A **mini quiz** on your first aid knowledge, whose anonymised data will promise to advance research.
- **A 30-minute E-learning session** to bring everything up to date
- Once you have completed the E-learning, you will be able to sign up for a **one-hour practice session** (you're not dreaming, even in COVID's time, we have managed to offer you a live practice session with real people!)
- And if you complete the E-learning and the training, **you will receive a certificate in BLS-AED** that will allow you to **register on the "Save a Life" network** and thus join the Geneva care system in advance!

All this is available at the following address: https://bls.anesth.ch
For questions: bls-aemg@unige.ch

Why participate?

- This will be your only opportunity to practice all year!
- You will discover the world of the emergency room for the first time
- To make fun of your friends in the higher years because you will be better trained in resuscitation than them
- The training is short
- The course is given by students from the higher years and networking never hurts
- You'll get a nice paper which is not only useful, but also useful on your CV
- To finally have a reason to put on some trousers and get out of your house

Basically, click on this link (https://bls.anesth.ch), go through the E-learning and come and learn how to save lives!

We look forward to seeing you,

Victor Taramarcaz & Tara Herren
