# Supplementary material for: A Short Intervention and an Interactive e-Learning Module to Motivate Medical and Dental Students to Enlist as First Responders: Implementation Study
Source: J Med Internet Res. 2022 May 18;24(5):e38508. doi: 10.2196/38508 (PMC9161047; doi:10.2196/38508)
Supplement: Multimedia Appendix 2 [file jmir_v24i5e38508_app2.pdf]

Multimedia appendix 2 – Screening questionnaire and consent form

N°	*Questions et réponses (FR)*	*Questions and answers (EN)*	
**1**	**Êtes-vous déjà first responder ?**<br><br>Question à choix multiple (une seule réponse) :<br>1. Oui<br>2. Non	**Are you already a first responder?**<br><br>Multiple choice question (only one possible answer):<br>1. Yes<br>2. No	Intended for all students
**2**	**Avez-vous déjà répondu à ce questionnaire ?**<br><br>Si vous avez déjà répondu à ces questions et avez déjà un compte utilisateur, cliquez ici	**Have you already filled this questionnaire?**<br><br>If you answered these questions already and have a user account, click here	Intended for all students
**3**	**Êtes-vous étudiant.e en médecine en 1ère année à l'Université de Genève**<br><br>Question à choix multiple (une seule réponse) :<br>1. Oui<br>2. Non	**Are you a first-year medical student?**<br><br>Multiple choice question (only one possible answer):<br>1. Yes<br>2. No	Intended for all students
**4**	**Quel est votre statut professionnel ?**<br><br>Texte libre	**What is your professional status?**<br><br>Free text	If no to the question 3
**5**	**Acceptez-vous de participer à cette étude/formation ?**<br><br>Question à choix multiple (une seule réponse) :<br>1. Oui<br>2. Non	**Do you accept to participate to this study/training?**<br><br>Multiple choice question (only one possible answer):<br>1. Yes<br>2. No	Intended for all students
**6**	**Quelles sont les raisons de votre refus ?**<br><br>Question à réponses multiples (≥ 1 réponse possible) :<br>1. Manque de temps<br>2. Sujet peu intéressant<br>3. Déjà formé aux manœuvres de réanimation<br>4. Déjà engagé comme first responder<br>5. Autre (texte libre)	**What are the reasons for your refusal?**<br><br>Multiple answer question (≥ 1 possible answer):<br>1. Lack of time<br>2. Not interesting topic<br>3. Already trained to resuscitation gesture<br>4. Already a first responder<br>5. Other (free text)	If no to the question 5

**7**	**Désirez-vous tout de même accéder au module e-learning ?**<br><br>Question à choix multiple (une seule réponse) :<br>1. Oui<br>2. Non	**Do you want to access the e-learning anyway ?**<br><br>Multiple choice question (only one possible answer):<br>1. Yes<br>2. No	If no to the question 5
