# Supplementary material for: A Short Intervention and an Interactive e-Learning Module to Motivate Medical and Dental Students to Enlist as First Responders: Implementation Study
Source: J Med Internet Res. 2022 May 18;24(5):e38508. doi: 10.2196/38508 (PMC9161047; doi:10.2196/38508)
Supplement: Multimedia Appendix 3 [file jmir_v24i5e38508_app3.pdf]

Multimedia appendix 3 – Initial page of the website

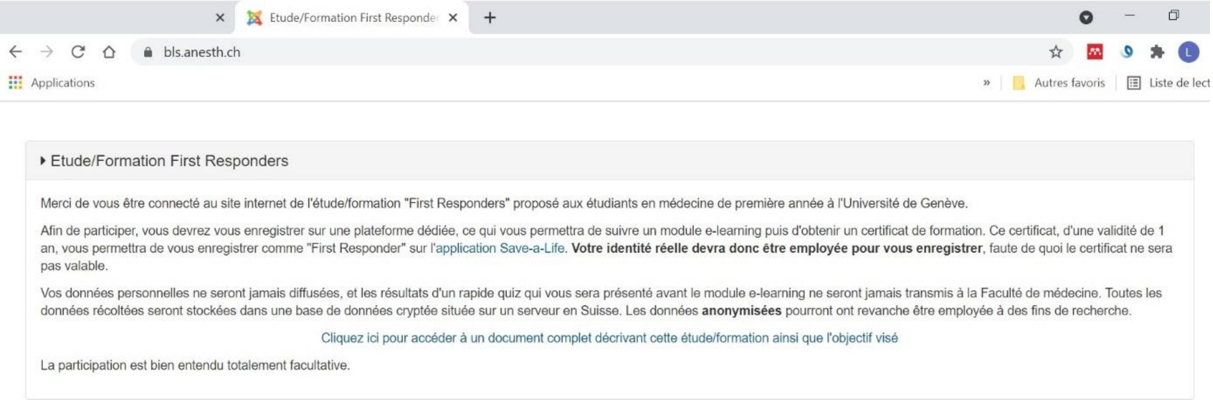

▸ Etude/Formation First Responders

Merci de vous être connecté au site internet de l'étude/formation "First Responders" proposé aux étudiants en médecine de première année à l'Université de Genève.

Afin de participer, vous devrez vous enregistrer sur une plateforme dédiée, ce qui vous permettra de suivre un module e-learning puis d'obtenir un certificat de formation. Ce certificat, d'une validité de 1 an, vous permettra de vous enregistrer comme "First Responder" sur l'application Save-a-Life. **Votre identité réelle devra donc être employée pour vous enregistrer**, faute de quoi le certificat ne sera pas valable.

Vos données personnelles ne seront jamais diffusées, et les résultats d'un rapide quiz qui vous sera présenté avant le module e-learning ne seront jamais transmis à la Faculté de médecine. Toutes les données récoltées seront stockées dans une base de données cryptée située sur un serveur en Suisse. Les données **anonymisées** pourront ont revanche être employée à des fins de recherche.

Cliquez ici pour accéder à un document complet décrivant cette étude/formation ainsi que l'objectif visé

La participation est bien entendu totalement facultative.
