# Supplementary material for: A Short Intervention and an Interactive e-Learning Module to Motivate Medical and Dental Students to Enlist as First Responders: Implementation Study
Source: J Med Internet Res. 2022 May 18;24(5):e38508. doi: 10.2196/38508 (PMC9161047; doi:10.2196/38508)
Supplement: Multimedia Appendix 4 [file jmir_v24i5e38508_app4.pdf]

INTEGRATION DES ETUDIANTS EN MÉDECINE EN 1ÈRE ANNÉE À L'UNIVERSITÉ DE GENÈVE À UN SYSTÈME DE « FIRST RESPONDERS »

CE PROJET EST ORGANISÉ ET SOUTENU PAR LES HÔPITAUX UNIVERSITAIRES DE GENÈVE ET PAR LA FACULTÉ DE MÉDECINE DE L'UNIVERSITÉ DE GENÈVE

Cher étudiant, chère étudiante,

Nous vous proposons de participer à une étude/formation dont vous trouverez les détails ci-dessous.

1. OBJECTIF DU PROJET

Notre but est de déterminer si un processus de formation rapide peut permettre à des étudiants en médecine en première année d'être intégrés à un réseau de « first responders », ou « premiers répondants ».

Les « first responders » sont des volontaires formés aux premiers secours, professionnels ou non professionnels de la santé, membres ou non de corps constitués (police, pompiers), qui acceptent d'être alarmés même en dehors de leurs heures de travail officielles en cas d'urgence médicale.

Le but principal du système de « first responders » est de diminuer le temps entre le moment de l'arrêt cardiaque et le début des manœuvres de réanimation, ce qui permet d'augmenter les chances de survie et améliore le pronostic neurologique des victimes.

2. SÉLECTION DES PERSONNES POUVANT PARTICIPER AU PROJET

La participation est ouverte à tous étudiants en médecine en 1ère année à l'Université de Genève.

3. SYSTÈME DE « FIRST RESPONDERS »

Des informations détaillées sur le système peuvent être consultées sur le site de Save-a Life : https://www.save-a-life.ch/.

4. CONSIDÉRATIONS ÉTHIQUES

Ce projet est conduit dans le respect des prescriptions légales helvétiques et internationales. La commission cantonale d'éthique a émis une déclaration de non-objection.

5. DÉROULEMENT POUR LES PARTICIPANTS

Vous avez suivi une brève présentation effectuée par des étudiants de l'Association des Etudiants en Médecine de Genève (AEMG) au début d'un cours, ce qui vous a conduit à accéder à ce site à l'aide de l'URL ou du code QR qui vous ont été fournis.

Vous êtes maintenant invités à déterminer, sur la base des informations qui vous ont été fournies, si vous désirez participer à ce projet.

Dans l'affirmative, vous devrez, après confirmé votre désir de participer, vous inscrire sur la plateforme en employant votre identité réelle. Cela est nécessaire pour qu'un certificat à votre nom puisse être généré par la suite. Après l'inscription, un rapide questionnaire visant à établir votre profil et à évaluer vos connaissances de base vous sera proposé. Vous suivrez ensuite un module e-learning adapté au contexte de la pandémie COVID-19, puis aurez la possibilité de vous enregistrer pour une session pratique. Les dates de ces sessions vous seront communiques par courriel. Ces sessions seront animées par des étudiants en médecine disposant d'un certificat d'instructeur validé selon les normes du Swiss Resuscitation Council. Après avoir réussi la partie pratique et obtenu votre certificat, vous pourrez vous enregistrer comme « first responder » sur la plateforme Save-a-Life. La validité du certificat obtenu est de 1 an.

Si vous refusez de participer, vous aurez tout de même la possibilité de suivre le module e-learning.

6. DROITS DES PARTICIPANTS

Vous êtes libre d'accepter ou de refuser de participer au projet. Si vous choisissez de ne pas participer ou si vous choisissez de participer et revenez sur votre décision pendant le déroulement du projet, vous n'aurez pas à vous justifier. Vous pouvez à tout moment poser toutes les questions nécessaires au sujet de l'étude auprès des investigateurs mentionnés à la fin de ce document.

7. CONFIDENTIALITÉ DES DONNÉES

Toutes les données seront stockées dans une base de donnée cryptée hébergée sur un serveur sécurisé localisé en Suisse.

Seul un investigateur aura accès à l'ensemble des données. Toutes les extractions de données seront anonymisées, et votre identité ne sera jamais accessible sur la base des données extraites. Les résultats du questionnaire initial seront transmis uniquement à des fins de recherche et seulement de manière anonymisée.

Dans le cas d'une publication, les données agrégées ne vous seront pas imputables en tant que personne. Votre nom n'apparaîtra jamais sur internet ou dans une publication. Toutes les personnes impliquées dans l'étude de quelque manière que ce soit sont tenues au secret professionnel. Toutes les directives relatives à la protection des données sont et seront respectées.

Il se peut que les données anonymes récoltées soient ultérieurement réutilisées à des fins d'analyse dans le cadre d'études scientifiques.

8. RÉMUNÉRATION DES PARTICIPANTS

Aucune rémunération ne sera octroyée pour votre participation.

Par votre participation, vous autorisez explicitement les investigateurs à utiliser les données anonymes obtenues à des fins de publication scientifique, et renoncez à toute requête de rétribution scientifique ou financière.

9. FINANCEMENT DU PROJET

L'étude est intégralement autofinancée, et n'est soutenue par aucun fond externe.

10. INTERLOCUTEUR(S)

Vous pouvez vous adresser à tout moment à l'un des interlocuteurs suivants :

Responsable du projet :

- Dr Laurent Suppan – laurent.suppan@hcuge.ch

Collaborateurs :

- Victor Taramarcaz – victor.tara@hotmail.com
- Tara Herren – Tara.Herren@etu.unige.ch
- Professeur Eduardo Schiffer – eduardo.schiffer@hcuge.ch
