# Supplementary material for: A Short Intervention and an Interactive e-Learning Module to Motivate Medical and Dental Students to Enlist as First Responders: Implementation Study
Source: J Med Internet Res. 2022 May 18;24(5):e38508. doi: 10.2196/38508 (PMC9161047; doi:10.2196/38508)
Supplement: Multimedia Appendix 5 [file jmir_v24i5e38508_app5.pdf]

Multimedia appendix 5 – Pre-course questionnaire

N°	*Questions et réponses (FR)*	Questions and answers (EN)
1	**Quelle est votre année de naissance ?**<br><br>Texte libre (Regex)	**What is your year of birth?**<br><br>Free text (Regex)
2	**Vous êtes :**<br><br>Question à choix multiple (une seule réponse) :<br>1. Une femme<br>2. Un homme<br>3. Autre	**You are:**<br><br>Multiple choice question (only one possible answer):<br>1. A woman<br>2. A man<br>3. Other
3	**Vous êtes étudiant.e. en médecine :**<br><br>Question à choix multiple (une seule réponse) :<br>1. Humaine<br>2. Dentaire<br>3. Autre<br>4. Je ne suis pas étudiant en médecine	**You are a student of medicine:**<br><br>Multiple choice question (only one possible answer):<br>1. Human<br>2. Dental<br>3. Other<br>4. I am not a medicine student
4	**Êtes-vous déjà un.e professionnel.le de la santé d'une autre profession médicale ou paramédicale**<br><br>Question à choix multiple (une seule réponse) :<br>1. Oui<br>2. Non	**Are you already a healthcare professional in a medical or paramedical field?**<br><br>Multiple choice question (only one possible answer):<br>1. Yes<br>2. No
5	**Quelle est cette profession ?**<br><br>Question à choix multiple (une seule réponse) :<br>1. Infirmier.e<br>2. Ambulancier.e<br>3. Sage-femme<br>4. Physiothérapeute<br>5. Pharmacien<br>6. Autre (texte libre)	**If yes, what is this profession?**<br><br>Multiple choice question (only one possible answer):<br>1. Nurse<br>2. Paramedic<br>3. Midwife<br>4. Physiotherapist<br>5. Pharmacist<br>6. Other (free text)
6	**Avez-vous déjà été étudiant.e. d'une autre profession médicale ou paramédicale ?**<br><br>Question à choix multiple (une seule réponse) :<br>1. Oui<br>2. Non	**Have you ever been a student in another medical or paramedical field?**<br><br>Multiple choice question (only one possible answer):<br>1. Yes<br>2. No

<table>
<tr><td>7</td><td>Laquelle ?

Question à choix multiple (une seule réponse) :
1. Infirmier.e
2. Ambulancier.e
3. Sage-femme
4. Physiothérapeute
5. Pharmacien
6. Autre (texte libre)</td><td>Which one ?

Multiple choice question (only one possible answer):
1. Nurse
2. Paramedic
3. Midwife
4. Physiotherapist
5. Pharmacist
6. Other (free text)</td></tr>
<tr><td>8</td><td>Pensez-vous déjà vous spécialiser dans un domaine particulier de la médecine ?

Question à choix multiple (une seule réponse) :
1. Oui
2. Non</td><td>Do you already know in which field of medicine you want to specialize?

Multiple choice question (only one possible answer):
1. Yes
2. No</td></tr>
<tr><td>9</td><td>Dans quel domaine en particulier ?

Question à choix multiple (une seule réponse) :
1. Cardiologie
2. Chirurgie
3. Gynécologie-obstétrique
4. Médecine interne (ou autre sous-spécialité hormis la cardiologie : pneumologie, néphrologie, etc.)
5. Médecine aigue (anesthésie, urgence, soins intensifs)
6. Neurosciences cliniques (neurologie, ophtalmologie, etc.)
7. Pédiatrie
8. Recherche fondamentale
9. Autre</td><td>In what field?

Multiple choice question (only one possible answer):
1. Cardiology
2. Surgery
3. Gynaecology-obstetric
4. Internal medicine (or subcategory except cardiology: pneumology, nephrology, etc.)
5. Acute medicine (anaesthesiology, emergency, intensive care)
6. Clinical neuroscience (neurology, ophthalmology, etc.)
7. Paediatrics
8. Fundamental research
9. Other</td></tr>
<tr><td>10</td><td>Avez-vous déjà entendu parler de BLS/ACLS ?

Question à choix multiple (une seule réponse) :
1. Oui
2. Non</td><td>Have you ever heard of BLS or ACLS?

Multiple choice question (only one possible answer):
1. Yes
2. No</td></tr>
<tr><td>11</td><td>Que veut dire « DSA » ?

Réponses acceptées (texte libre) : toute réponse contenant le mot « défibrillateur » sans tenir compte de la casse ou de faute de frappes.</td><td>What does « AED » mean?

Answers accepted (free text): all answers containing the word “defibrillator” regardless of how it was spelled</td></tr>
<tr><td>12</td><td>En quelle année les guidelines de réanimation ont-elles été révisées pour la dernière fois ?

Réponse attendue (texte libre) : 2015</td><td>In what year were the resuscitation guideline last revised?

Expected answer (free text): 2015</td></tr>
</table>

**13**	**Quel est le numéro à appeler en cas d'urgences médicales ?**<br><br>*Réponse acceptées (texte libre) : 144, 112, 911*	**What is the phone number for medical emergency?**<br><br>*Accepted answer (free text): 144, 112, 911*
**14**	**Quel est votre niveau d'entrainement à la réanimation ?**<br><br>Question à choix multiple (une seule réponse) :<br>1. Je n'ai jamais été entraîné<br>2. J'ai suivi un seul cours de réanimation<br>3. J'ai suivi plusieurs cours de réanimation<br>4. Je suis instructeur/instructrice BLS<br>5. Je suis sauveteur professionnel	**What is your training level for resuscitation?**<br><br>Multiple choice question (only one possible answer):<br>1. I have never been trained<br>2. I have followed only one resuscitation course<br>3. I have followed multiple resuscitation course<br>4. I am a BLS instructor<br>5. I am a professional rescuer
**15**	**Pour quelle(s) raison(s) n'avez-vous jamais suivi une telle formation auparavant ?**<br><br>Question à réponses multiples (≥ 1 réponse possible) :<br>1. On ne me l'a pas proposé<br>2. Je ne pense pas que cela soit important<br>3. Mon emploi du temps est trop chargé<br>4. La formation est trop couteuse<br>5. Autre (texte libre)	**For what reason(s) have you never taken such training before?**<br><br>Multiple answer question (≥ 1 possible answer):<br>1. I have never been offered<br>2. I do not think that is important<br>3. My work schedule is full<br>4. Trainings are too expensive<br>5. Other
**16**	**Voudriez-vous davantage d'entrainement à la réanimation ?**<br><br>Question à choix multiple (une seule réponse) :<br>1. Oui<br>2. Non	**Would you like to have more resuscitation?**<br><br>Multiple choice question (only one possible answer):<br>1. Yes<br>2. No
**17**	**Quels sont les critères qui doivent être présents pour affirmer qu'un patient est en ACR**<br><br>Question à réponses multiples (≥ 1 réponse possible) :<br>1. Absence de réponse à la stimulation verbale et tactile<br>2. Langage désorganisé (ou incohérent)<br>3. Respiration absente ou anormale (lente et laborieuse)<br>4. Absence de pouls périphérique<br>5. Absence de pouls central<br><br>*Réponses attendues : 1 et 3*	**What are the criteria to confirm that a patient is in cardiac Arrest?**<br><br>Multiple answer question (≥ 1 possible answer):<br>1. Lack of response to verbal and tactile stimuli<br>2. Disorganised speech (or incoherent)<br>3. Lack of breathing or abnormal (slow and difficult)<br>4. Lack of peripheral pulse<br>5. Lack of central pulse<br><br>*Expected answers: 1 and 3*

**18**	**Pour traiter un patient en arrêt cardiorespiratoire, dans quel ordre procédez-vous**<br><br>Mettre dans l'ordre :<br>1. Libération des voies aériennes<br>2. Insufflations<br>3. Compressions thoraciques<br><br>*Réponse attendue : 3-1-2*	**To treat a patient that is in cardiac arrest, in which order do you proceed?**<br><br>Ordering<br>1. Airway release<br>2. Ventilation<br>3. Thoracic compressions<br><br>*Expected answer: 3-1-2*
**19**	**Quelle est l'artère idéale pour ressenti un pouls chez un patient adulte ?**<br><br>Question à choix multiple (une seule réponse) :<br>1. L'artère carotide<br>2. L'artère brachiale<br>3. L'artère radiale<br>4. L'artère fémorale<br><br>*Réponse attendue : 1*	**What is the ideal artery to feel the pulse on an adult patient?**<br><br>Multiple choice question (only one possible answer):<br>1. Carotid artery<br>2. Brachial artery<br>3. Radial artery<br>4. Femoral Artery<br><br>Expected answer: 1
**20**	**Lors du massage cardiaque, quelle est la profondeur idéale à obtenir chez un adulte ?**<br><br>Question à choix multiple (une seule réponse) :<br>1. 2-3 cm<br>2. 3-4 cm<br>3. 4-5 cm<br>4. 5-6 cm<br>5. 6-7 cm<br><br>*Réponse attendue : 4*	**During a cardiac massage, what is the ideal depth to obtain in an adult?**<br><br>Multiple choice question (only one possible answer):<br>1. 2-3 cm<br>2. 3-4 cm<br>3. 4-5 cm<br>4. 5-6 cm<br>5. 6-7 cm<br><br>Expected answer : 4
**21**	**Si on pratique la ventilation artificielle en parallèle du massage cardiaque, quel est le ratio compressions :insufflations à effectuer**<br><br>Question à choix multiple (une seule réponse) :<br>1. 15 :2<br>2. 25 : 2<br>3. 30 :1<br>4. 30 : 2<br>5. 60 :4<br><br>*Réponse attendue : 4*	**If we practice artificial ventilation with cardiac massage, what is the compression/insufflations ratio to use?**<br><br>Multiple choice question (only one possible answer):<br>1. 15 :2<br>2. 25 : 2<br>3. 30 :1<br>4. 30 : 2<br>5. 60 :4<br><br>*Expected answer : 4*

**22**	**À quelle fréquence les compressions doivent-elles être effectuées ?**<br><br>Question à choix multiple (une seule réponse) :<br>1. 60-80 compressions par minute<br>2. 80-100 compressions par minute<br>3. 100-120 compressions par minute<br>4. 120-140 compressions par minute<br>5. 140-160 compressions par minute<br><br>*Réponse attendue : 3*	**At what frequency do the compressions have to be performed at?**<br><br>Multiple choice question (only one possible answer):<br>1. 60-80 compressions per minute<br>2. 80-100 compressions per minute<br>3. 100-120 compressions per minute<br>4. 120-140 compressions per minute<br>5. 140-160 compressions per minute<br><br>*Expected answer : 3*
**23**	**Si aucune insufflation n'est administrée, les compressions thoraciques restent-elles utiles ?**<br><br>Question à choix multiple (une seule réponse) :<br>1. Oui<br>2. Non<br>3. Je ne sais pas	**If no insufflation is administered, are the thoracic compressions still relevant?**<br><br>Multiple choice question (only one possible answer):<br>1. Yes<br>2. No<br>3. I do not know
**24**	**Quelle est la première action recommandée face à un patient adulte qui s'étouffe, sachant qu'il est incapable de parler ni de tousser ?**<br><br>Question à choix multiple (une seule réponse) :<br>1. Commencer une réanimation cardiopulmonaire<br>2. Je demande quand même au patient d'essayer de tousser<br>3. Tenter une manœuvre de Heimlich<br>4. Aller chercher l'obstacle dans la bouche du patient<br><br>*Réponse attendue : 3*	**What is the first action recommended in a situation where an adult patient is choking, knowing he is incapable to speak or cough?**<br><br>Multiple choice question (only one possible answer):<br>1. Begin a cardiopulmonary resuscitation<br>2. Ask the patient to cough anyway<br>3. Try a Heimlich manoeuvre<br>4. Try to catch the obstacle in the patient's mouth<br><br>Expected answer: 3
**25**	**Sur une échelle de 1 à 10, comment qualifieriez-vous votre aisance face à une situation de réanimation ?**	**On a scale of 1 to 10, how would you rate your confidence in a resuscitation situation?**
