# Supplementary material for: A Short Intervention and an Interactive e-Learning Module to Motivate Medical and Dental Students to Enlist as First Responders: Implementation Study
Source: J Med Internet Res. 2022 May 18;24(5):e38508. doi: 10.2196/38508 (PMC9161047; doi:10.2196/38508)
Supplement: Multimedia Appendix 6 [file jmir_v24i5e38508_app6.pdf]

1 *Multimedia appendix 6 – Post-course questionnaire*

N°	***Questions et réponses***	**Questions and answers**	
**1**	**Avez-vous apprécié cette formation ?**<br><br>Question à choix multiple (une seule réponse) :<br>1. Oui<br>2. Non	**Have you appreciated this training?**<br><br>Multiple choice question (only one possible answer):<br>1. Yes<br>2. No	Intended for all students
**2**	**Vous avez trouvé la formation :**<br><br>Question à réponses multiples (≥ 1 réponse possible) :<br>1. Utile<br>2. Intéressante<br>3. Bien expliquée<br>4. Captivante<br>5. Une bonne occasion de faire de la pratique	**You thought the training:**<br><br>Multiple answer question (≥ 1 possible answer):<br>1. Useful<br>2. Interesting<br>3. Well explained<br>4. Captivating<br>5. A good occasion for practice	If yes to question 1
**3**	**Vous avez trouvé la formation :**<br><br>Question à réponses multiples (≥ 1 réponse possible) :<br>1. Répétitive<br>2. Trop semblable au cours pour le permis de conduire<br>3. Pas instructive<br>4. Trop courte<br>5. Inintéressante	**You thought the training:**<br><br>Multiple answer question (≥ 1 possible answer):<br>1. Repetitive<br>2. Too similar to the driving licence resuscitation course<br>3. Not informative<br>4. Too short<br>5. Not interesting	If no to question 1
**4**	**Avez-vous d'autres commentaires à formuler par rapport à l'intérêt de cette formation ?**<br><br>Texte libre	**Do you have any other comment regarding the interest of this training?**<br><br>Free text	Intended for all students
**5**	**Était-ce votre première formation en premier secours ?**<br><br>Question à choix multiple (une seule réponse) :<br>1. Oui<br>2. Non	**Was it your first aid training?**<br><br>Multiple choice question (only one possible answer):<br>1. Yes<br>2. No	Intended for all students
**6**	**Quel(s) cours aviez-vous déjà effectué(s) ?**<br><br>Question à réponses multiples (≥ 1 réponse possible) :<br>1. Permis de conduire<br>2. Samaritains<br>3. Armée<br>4. Autre (texte libre)	**What course have you already done?**<br><br>Multiple answer question (≥ 1 possible answer):<br>1. Driver licence<br>2. Samaritan<br>3. Army<br>4. Other (free text)	Intended for all students

<table>
<tr><td>7</td><td>Avant de suivre ce cours, vous sentiez-vous à l’aise à l’idée d’intervenir en cas d’arrêt cardiaque en dehors de l’hôpital ?

Échelle de Likert :
1. Absolument pas
2.
3.
4.
5. Totalement</td><td>Before taking this course, were you comfortable with the idea of responding to a cardiac arrest outside of the hospital?

Likert scale:
1. Absolutely not
2.
3.
4.
5. Totally</td><td>Intended for all students</td></tr>
<tr><td>8</td><td>Et maintenant ?

Échelle de Likert :
1. Absolument pas
2.
3.
4.
5. Totalement</td><td>And now?

Likert scale:
1. Absolutely not
2.
3.
4.
5. Totally</td><td>Intended for all students</td></tr>
<tr><td>9</td><td>Les facteurs suivants ont contribué à l’augmentation de votre degré de confiance :

Échelle de Likert :
1. Meilleure connaissance du sujet (1-5)
2. Sentiment de maitriser le sujet (1-5)
3. Meilleure compréhension des enjeux de santé (1-5)</td><td>Fallowing factors helped to increase your confidence

Likert scale:
1. Better knowledge on the subject (1-5)
2. Feeling to master the subject (1-5)
3. Better understanding of health issues (1-5)</td><td>Intended for all students</td></tr>
<tr><td>10</td><td>Les facteurs suivants limitent votre confiance en votre aptitude à réanimer :

Échelle de Likert :
1. Peur de faire faux (1-5)
2. Stress (1-5)
3. Sentiment de ne pas en être capable (1-5)</td><td>Fallowing factors are limiting your confidence to perform a resuscitation:

Likert scale:
1. Fear to do wrong (1-5)
2. Stress (1-5)
3. Feeling that I am not capable of (1-5)</td><td>Intended for all students</td></tr>
<tr><td>11</td><td>Avez-vous d’autres remarques à formuler par rapport à votre sentiment de confiance en votre aptitude à réanimer ?

Texte libre</td><td>Have you any other comment about your feeling of confidence in your ability to perform a resuscitation

Free text</td><td>Intended for all students</td></tr>
<tr><td>12</td><td>Avez-vous l’intention de vous inscrire sur l’application « Save a Life » en tant que first responder ?

Question à choix multiple (une seule réponse) :
1. Oui
2. Non</td><td>Do you intend to register on the “Save a Life” app as a first responder?

Multiple choice question (only one possible answer):
1. Yes
2. No</td><td>Intended for all students</td></tr>
</table>

**13**	**Quels sont les facteurs qui ont contribué à votre décision ?**<br><br>Échelle de Likert :<br>1. Envie d'aider (1-5)<br>2. Les enjeux sont importants (1-5)<br>3. Sentiment de pouvoir faire la différence (1-5)<br>4. Je me sens maintenant capable d'intervenir (1-5)	**What are the factors that contributed to your decision?**<br><br>Likert scale:<br>1. Envy to help (1-5)<br>2. The stakes are important (1-5)<br>3. Feeling to be able to make de difference (1-5)<br>4. I feel now able to deal with it	If yes to question 12
**14**	**Quels sont les facteurs qui vous retiennent de vous inscrire ?**<br><br>Échelle de Likert :<br>1. Pas envie de participer à ce programme (1-5)<br>2. Trop stressant (1-5)<br>3. Impression que c'est inutile (1-5)<br>4. Je ne me sens pas capable d'intervenir (1-5)	**What are the factors that are keeping you to sign up?**<br><br>Likert scale:<br>1. I do not want to participate to this program (1-5)<br>2. Too stressful (1-5)<br>3. Feeling that it is not useful (1-5)<br>4. I do not feel able to deal with it (1-5)	If no to question 12
**15**	**D'autres facteurs sont-ils entrés en ligne de compte ?**<br><br>Texte libre	**Did other factors intervene in your decision?**<br><br>Free text	Intended for all students
**16**	**Recommanderiez-vous cette formation à d'autres étudiant.e.s en médecine ?**<br><br>Question à choix multiple (une seule réponse) :<br>1. Oui<br>2. Non	**Would you recommend this training to other medicine students?**<br><br>Multiple choice question (only one possible answer):<br>1. Yes<br>2. No	Intended for all students
**17**	**Auriez-vous des améliorations à nous proposer ?**<br><br>Texte libre	**Have you any other suggestion to make?**<br><br>Free text	Intended for all students
